# Medication beliefs and use of medication lists – is there a connection? Results from a before-and-after study in Germany

**DOI:** 10.1186/s12877-020-01513-y

**Published:** 2020-03-30

**Authors:** Cornelia Straßner, Cornelia Mahler, Beate Strauß, Ulrich Wehrmann, Katja Krug, Joachim Szecsenyi, Walter Emil Haefeli, Hanna Marita Seidling

**Affiliations:** 1 Department of General Practice and Health Services Research, University Hospital Heidelberg, Im Neuenheimer Feld 130.3, 69120 Heidelberg, Germany; 2 Department of Nursing Science, University Hospital Tuebingen, Institute of Health Sciences, Hoppe-Seyler-Str. 9, 72076 Tuebingen, Germany; 3 Berchelmann’sche Apotheke, Eberstädter Str. 63, 64319 Pfungstadt, Germany; 4 OCM-Consult, Bayernstr. 56, 67061 Ludwigshafen, Germany; 5 Department of Clinical Pharmacology and Pharmacoepidemiology, University Hospital Heidelberg, Im Neuenheimer Feld 410, 69120 Heidelberg, Germany

**Keywords:** Medication beliefs, Medication list, Medication reconciliation, Over-the-counter drugs, Behaviour change, Campaign

## Abstract

**Background:**

Despite increasing digitalisation the paper-based medication list remains one of the most important instruments for the documentation and exchange of medication-related information. However, even elderly patients with polypharmacy who are at high risk for medication errors and adverse drug events, frequently do not receive or use a complete and comprehensible medication list. Increasing the use of medication lists would be a great contribution to medication safety and facilitate the work of health care providers.

**Methods:**

This study is related to the project *MeinPlan* (MyPlan) which comprised an information campaign on safe drug administration in the Rhine-Neckar region in South Germany. The campaign was evaluated in a before-and-after study based on a survey among two independent, representative samples of citizens over 65 years. In total, 5034 questionnaires were analysed. While the effects of the primary outcome (the percentage of citizens using a medication list) have been reported elsewhere, this analysis focusses on the effects of the campaign on citizens’ medication beliefs and assesses whether medication beliefs are associated with the use of medication lists, the use of over-the-counter drugs and the use of the tools offered by the campaign. Medication beliefs were assessed with the German version of the General Beliefs About Medicines Questionnaire (BMQ) which results in subscales for “General Overuse”, “General Usefulness” and “General Harm”. The use of medication lists and over-the-counter drugs was assessed with self-developed questionnaire items.

**Results:**

No statistically significant change in citizens’ medication beliefs before and after the campaign could be detected. Likewise, no association between medication beliefs and the use of medication lists, the use of over-the-counter drugs or the use of the tools offered by the campaign could be shown.

**Conclusions:**

A campaign focussing on the risks of drug administration did not change the medication beliefs of the targeted population. Moreover, citizens’ general medication beliefs do not seem to be crucial for their decision to use a medication list or over-the-counter drugs. Strategies to improve the use of medication lists by patients should focus on other influential factors, such as individual benefits and barriers and socio-psychological factors.

## Background

Prescribing medication is one of the most frequently applied measures to treat or prevent diseases. Across Europe, 31% of the older adults take 5 drugs or more per day [1]. With the number of prescribed drugs, the risk of hospitalisation due to adverse drug reactions, mainly falls, fractures, infections and bleedings, also increases [2]. Drug therapy is a complex and therefore error-prone process comprising the recommendation for a certain medication which should be based on the inventory of a patient’s current medication as well as the assessment of the individual’s preferences and needs, the writing of the prescription, the dispensing of the drug e.g. in a pharmacy, the application of the drug by the patient and the monitoring of the treatment effects [3].

In general, many persons and institutions are involved in this process. In Germany, adults over 60 years have an average of 11.9 ambulatory doctor’s appointments per year contacting specialists in 4.2 different fields and spending, on average, 10.7 days in hospital each year [4]. Beside prescribers in inpatient and outpatient care, other professions, such as pharmacists, nurses as well as informal care givers, might also be involved in the process of care and drug administration.

To ensure efficient communication among all these players and to support patients in correct drug administration, timely, accurate and comprehensible documentation of the essential medication-related information is crucial. Despite ongoing attempts to establish electronic medical records, to-date, the paper-based medication list is the most common instrument for this purpose in Germany and in 2016 – 2 years after conduction of the *MeinPlan (MyPlan)* campaign – patients’ right to receive a medication list became codified law.

However, not all patients on long-term medication have a medication list. If they do, discrepancies between the medication documented and the medication actually taken by the patients have been shown in over 70% of the cases [5–8] while 30% of these discrepancies are also considered potentially harmful [9]. Furthermore, medication lists are frequently not available when needed because patients do not always carry them along or they use their own handwritten medication lists which lack important information or are illegible [10]. On the other hand, medication lists issued by health care professionals may not meet patients’ needs because they list information relevant to other health care professionals rather than giving adequate information to facilitate patients’ self-support. For instance, in a study examining patients’ understanding of a medication list, 35% of the participants were not satisfied with the design and 50% misunderstood the abbreviations given on the list [11].

The aim of the project *MeinPlan* conducted by the “Aktionsbündnis Sichere Arzneimitteltherapie Heidelberg/Rhein-Neckar” (Active Alliance for Safe Pharmacotherapy Heidelberg/Rhine-Neckar) was to raise awareness among the elderly population of the Rhine-Neckar region in South Germany about avoidable risks of drug treatment as well as possibilities for citizens to actively contribute to a safe drug treatment. Informing health care providers about the medication they actually take and avoiding administration errors were considered important contributions. Since an updated and comprehensible medication list is a useful tool for these purposes, the main objective was to increase the percentage of citizens on long-term medication using a medication list. The effects of the project were examined in a before-and-after study based on a representative survey.

**Fig. 1:**
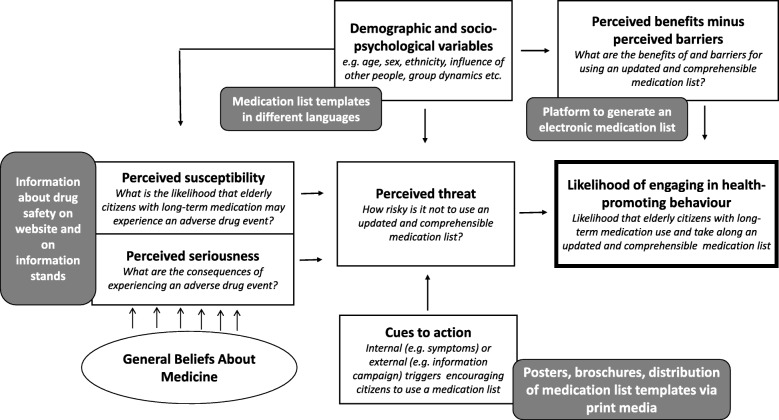
Health Belief Model, modified according to Janz et al. 1984 [12]. The figure demonstrates how the elements of the *MeinPlan* intervention (grey boxes) are related to the Health Belief Model (white boxes). We assumed that general beliefs about medicines influence the risk awareness concerning polypharmacy. The hypothesis was that cizitens with a rather positive attitude towards medications are less risk-conscious and therefore less likely to use a medication list

According to the Health Belief Model [12], several factors influence the likelihood of engaging in a health-related behaviour. Figure 1 shows which strategies were used in the *MeinPlan* project and how they are related to the Health Belief Model. A major focus of the project was the citizens’ perception of the risks coming with polypharmacy. Hence, we intended to raise awareness in this field. One hypothesis was that citizens’ general medication beliefs influence their risk awareness and therefore their readiness to use a medication list or to engage in other medication-related health behaviours. The Beliefs About Medicine Questionnaire (BMQ) measures medication beliefs and distinguishes three dimensions: *general harm, general usefulness* and *general overuse*, ascertaining whether respondents believe medications to be generally rather harmful or useful and whether there is a general overuse of medication in the society. We assumed that citizens with a rather positive attitude towards medication use (i.e. generating high scores for general usefulness and low scores for general harm and general overuse) would be less risk-conscious and therefore less likely to use a medication list. In addition, we were interested whether the *MeinPlan* project had an influence on citizens’ medication beliefs or not. Since we intended to raise awareness of the risks of polypharmacy, we assumed that the scores for general harm and general overuse might possibly raise.

While the descriptive results of the survey and the primary outcome of the study (percentage of citizens using a medication list) have been reported elsewhere [13], this article focusses on the following research questions:

1. Is there a difference regarding citizens’ medication beliefs before and after the campaign of the project *MeinPlan*?
2. Does a correlation/association exist between citizens’ medication beliefs and
  2.1 … the number of drugs taken?
  2.2 … the use of over-the-counter drugs?
  2.3 …the presence of a medication list?
  2.4 … the way the medication list is used?
  2.5 … the use of the tools (i.e. the website, the paper-based medication list template and the electronic medication list) offered by the project *MeinPlan*?

## Methods

### Study design

We conducted a non-randomised before-and-after study based on a written survey among two independent, representative samples of citizens aged over 65 years living in the Rhine-Neckar region of Southern Germany. The ethical approval was obtained from the responsible Ethics Committee of the Medical Faculty of Heidelberg University (number S-647/2013).

### Intervention

Within the scope of the project *MeinPlan,* several population-based interventions were conducted between June 2014 and December 2015 with the aim to raise the public’s and health care professionals’ awareness for the risks of pharmacotherapy and thus the importance of medication lists and correct administration of medication. The campaign comprised the distribution of about 60.000 medication list templates via the local press. Lectures were held in adult education centres and during public events for senior citizens. The website *www.nimmsrichtig.de* contributed teaching videos about safe medication administration, enabled to download and print off medication list templates in different languages and provided access to an internet platform allowing to generate and manage an electronic medication list with illustrated administration advices. In addition, posters, flyers and brochures were distributed to pharmacies and general practitioners in the region. More details about the interventions have been depicted in the main report of the study [13].

### Participants and setting

The target group of the survey were citizens aged 65 or above living in the Rhine-Neckar Region and the city of Heidelberg. The samples for the baseline and follow-up survey were independent, i.e. the individuals who participated in the first survey were excluded from the second one. Participants were randomly selected by an independent state institution. People living in a residential or nursing home were excluded by address.

### Variables

The questionnaires used at baseline and follow-up were largely identical and consisted both of the German version of the General Beliefs About Medicine Questionnaire (*BMQ General*) [14, 15] and non-validated but pre-tested, self-developed items. The *BMQ General* assesses medication beliefs in general (not necessarily related to the medication of the individual respondent) and consists of 12 items which form 3 sub-scales: *general harm, general usefulness* and *general overuse.* All items are rated on a five-point Likert scale (5 = strongly agree, 1 = strongly disagree). Sub-scale scores are calculated leading to values between 4 and 20. Higher scores indicate stronger belief in the respective concept, i.e. the general belief that there is an overuse of medication in the society and the general belief that medications are predominantly either harmful or useful. The German version of the *BMQ General* has proven to be a valid and reliable instrument with high internal consistency [15].

Alongside socio-demographic data, the self-developed items collected information on the number of drugs respondents took regularly, by whom these drugs were recommended or prescribed (physician, pharmacist, other), whether or not the respondent had support with drug administration (yes/no), and whether and how he or she used a medication list (updated regularly, shown when visiting a doctor or a pharmacy, taken along during emergencies or used as a reminder). At follow-up, four additional questions related to the *MeinPlan* campaign were posed.

### Data collection

Pseudonymised questionnaires were sent out by mail before the campaign started in June 2014 (T0) and after the end of the campaign in January 2016 (T1). To ensure confidentiality, the key for pseudonymisation was only available to the local health authority that organized the postal delivery of the questionnaires and not to Heidelberg University Hospital which received the responses and analysed the data. After 4 weeks, a reminder was sent. As an incentive, respondents were offered to participate in a price draw to win a book on medication administration.

### Sample size

Power calculation was performed for the primary end point, the number of people with a medication schedule, yielding a sample size of 5340 citizens needed per time point. Given the population of the Rhine-Neckar-region and Heidelberg, this referred to about 4% of the population 65 years or older.

### Data analysis and statistical methods

As a first step, descriptive analyses were conducted to describe the sample and to check for plausibility of the database. Implausible specifications mainly related to filter questions (respondents stating not to take any medication or not to have a medication list but answering all subsequent questions) were recoded and treated as missing values.

The t-test was used to determine the difference in medication beliefs as measured by the *BMQ General* at T0 and T1 (research question 1). For correlation analyses (research questions 2.1–2.5) the data sets of T0 and T1 were pooled. The Eta coefficient was determined for correlations between interval-scaled and categorical variables and the Spearman coefficient for correlations between two ordinal variables and between ordinal and interval-scaled variables. *P*-values < 0.05 were considered statistically significant, coefficients > 0.5 were regarded clinically relevant correlations.

## Results

### Participants

We received 2640 questionnaires in the baseline survey (response rate 49.4%) and 2427 questionnaires (response rate 45.4%) in the follow-up survey after 18 months. In total, 33 questionnaires were excluded because 17 were not filled in and because in 16 other cases respondents were younger than 65 years according to the indicated year of birth. This resulted in a total sample size of 5034 respondents.

Table 1 shows the characteristics of the participants. On average, respondents were 75 (65–101) years old with equal proportions of male (49.3%) and female (50.7%). The vast majority (90.6%, *N* = 4392) stated to take at least 1 drug regularly, 15.2% (*N* = 739) reported extensive polypharmacy with more than 7 drugs. Concerning the medication beliefs, *general usefulness* averaged higher than *general harm* (16.1 / SD 2.48 versus 9.5 / SD 2.97) suggesting that the average population of the survey considered drugs in general more beneficial than harmful. The mean for *general overuse* was 13.0 (SD 3.11) indicating that the average population of the survey was undecided whether there was general overuse of drugs in the society or not.

**Table 1:** Characteristics of the participants and descriptive results [absolute numbers are previously reported in [13]]

	T0	T1	T0 + T1
Total population	*N* = 2609	*N* = 2425	*N* = 5034
Age MEAN (RANGE)	73.9 (65–101) *N* = 2577	75.1 (65–100) *N* = 2376	74.5 (65–101) *N* =4953
Female PERCENTAGE (N/Ntotal)	51.6 (1334/2584)	49.6 (1182/2382)	50.7 (2516/4966)
Non-native German speakers PERCENTAGE (N/Ntotal)	3.8 (96/2517)	4.1 (93/2273)	3.9 (189/4790)
Population with regular medication intake PERCENTAGE (N/Ntotal)^a^	90.4 (2288/2530)	90.8 (2107/2320)	90.6 (4395/4850)
Number of medications taken			
0	9.6 (242/2530)	9.2 (213/2320)	9.4 (455/4850)
1–3	36.8 (931/2530)	36.8 (853/2320)	36.8 (1784/4850)
4–7	38.8 (982/2530)	38.3 (890/2320)	38.6 (1872/4850)
> 7	14.8 (375/2530)	15.7 (364/2320)	15.2 (739/4850)
Use of over-the-counter drugs PERCENTAGE (N/Ntotal) ^b^	27.7 (631/2279)	26.9 (563/2095)	27.2 (1194/4371)
Having a medication list PERCENTAGE (N/Ntotal) referred to all patients indicating regular drug intake ^a^	51.6 (1181/2288)	51.4 (1082/2107)	51.5 (2263/4395)
Last medication list up-date ^d^			
More than one year ago	30.7 (346/1127)	33.0 (345/1046)	31.8 (691/2173)
Less than one year ago	51.0 (575/1127)	51.0 (533/1046)	51.0 (1108/2173)
Never	5.6 (63/1127)	4.4 (46/1046)	5.0 (109/2173)
Never because medication has not changed	12.7 (143/1127)	11.7 (122/1046)	12.2 (265/2173)
Documenting self-medication, i.e. additional drugs purchased in the pharmacy or in the supermarket) on the medication list PERCENTAGE (N/Ntotal) ^c^	22.5 (266/1181)	21.6 (234/1082)	22.1 (500/2263)
Showing the medication list during doctor’s appointments PERCENTAGE (N/Ntotal)^a^	35.3 (417/1181)	36.7 (397/1082)	36.0 (814/2263)
Showing the medication list in the pharmacy PERCENTAGE (N/Ntotal) ^a^	2.5 (29/1181)	2.7 (29/1082)	2.6 (58/2263)
Taking the medication lists along for cases of emergency PERCENTAGE (N/Ntotal) ^a^	56.6 (669/1181)	58.0 (628/1082)	57.3 (1297/2263)
Using the medication list as reminder PERCENTAGE (N/Ntotal) ^a^	52.0 (614/1181)	55.2 (597/1082)	53,5 (1211/2263)
Not using the medication list at all PERCENTAGE (N/Ntotal) ^a^	16.9 (199/1181)	14.9 (161/1082)	15.9 (360/2263)
Having known the *MeinPlan* project before participating in the survey PERCENTAGE (N/Ntotal)^a^	Not applicable	5.7 (139/2425)	Not applicable
Having used the website www.nimmsrichtig.de PERCENTAGE (N/Ntotal) ^a^	Not applicable	1.3 (32/2425)	Not applicable
Having a used a medication list template provided by *MeinPlan* PERCENTAGE (N/Ntotal) ^a^	Not applicable	1.0 (25/2425)	Not applicable
Having used the electronic medication list provided by *MeinPlan* PERCENTAGE (N/Ntotal) ^a^	Not applicable	0.2 (5/2425)	Not applicable
BMQ subscale “General Overuse” MEAN (RANGE; SD)	13.0 (4–20; 3.06); *N* = 2428	12.9 (4–20; 3.16); *N* = 2260	13.0 (4–20; 3.11); *N* = 4688
BMQ subscale “General Usefulness” MEAN (RANGE; SD)	16.1 (4–20; 2.50); *N* = 2451	16.0 (4–20; 2.45); *N* = 2249	16.1 (4–20; 2.48); *N* = 4700
BMQ subscale “General harm” MEAN (RANGE; SD)	9.5 (4–20; 2.98); *N* = 2377	9.5 (4–20; 2.95); *N* = 2193	9.5 (4–20; 2.97); *N* = 4570

*N* number, *Ntotal* number of total responses (differences to the total population are due to missing values)

*SD* standard deviation

^a^Dichotomous variable (response categories yes/no). Numbers refer to the response category “yes”

^b^Dichotomous variable (response categories yes/no) calculated by summation of the variable “Use of drugs recommended by pharmacists without prescription” and “Use of drugs bought by myself in the supermarket / drug store”. Numbers refer to the response category “yes”

^c^Dichotomous variable (response categories yes/no) calculated by summation of the variable “I document medications bought in the pharmacy on my medication list” and “I document medications bought in the supermarket / drug store on my medication list”. Numbers refer to the response category “yes”

### Main results

Research question 1 focussed on whether there was a difference regarding the medication beliefs in the population before and after the *MeinPlan* campaign was conducted. The t-test showed no significant difference for all three sub-scales (see Table 2).

**Table 2:** Difference in the three sub-scales of the BMQ General between T0 and T1 (research question 1)

	*p*-value (t-test)	Confidence Interval	Mean difference
BMQ subscale “General Overuse”	0.389	- 0.100 – 0.256	0.078
BMQ subscale “General Usefulness”	0.156	- 0.039 – 0.244	0.103
BMQ subscale “General Harm”	0.774	- 0.147– 0.197	0.025

Table 3 shows the results of the correlation analyses. To answer research question 2.1, we examined whether there was an association between medication beliefs and the number of drugs regularly taken by the respondent. The Spearman correlation showed no clinically relevant relationship.

**Table 3:** Correlation analyses (research question 2.2–2.5)

Theme in the questionnaire ^a^		BMQ subscale “General Overuse”	BMQ subscale “General Usefulness”	BMQ subscale “General Harm”
Number of drugs taken	N	4564	4574	4449
	Spearman CC	−0.169	0.128	- 0.165
Use of over-the-counter drugs	N	4124	4132	4020
	Eta CC	0.043	0.040	0.027
Existence of a medication list	N	4049	4060	3948
	Eta CC	0.122	0.103	0.102
Last medication list up-date	N	3740	3733	3647
	Eta CC	0.121	0.096	0.093
Documentation of self-medication on the medication list	N	2106	2124	2064
	Eta CC	0.011	0.026	0.011
Showing the medication list during doctor’s appointments	N	2106	2124	2064
	Eta CC	0.003	0.011	0.009
Showing the medication list in the pharmacy	N	2106	2124	2064
	Eta CC	0.071	0.039	0.054
Taking the medication list along during emergencies	N	2106	2124	2064
	Eta CC	0.036	0.049	0.020
Use of the medication list as a reminder	N	2106	2124	2064
	Eta CC	0.039	0.006	0.004
No use of the medication list	N	2106	2124	2064
	Eta CC	0.036	0.008	0.010
Use of the website www.nimmsrichtig.de	N	2199	2188	2137
	Eta CC	0.021	0.000	0.023
Use of a medication list provided by the project *MeinPlan*	N	2196	2183	2131
	Eta CC	0.010	0.034	0.016
Use of the electronic medication list provided by the project *MeinPlan*	N	1412	1402	1369
	Eta CC	0.027	0.001	0.017

^a^*CC* correlation coefficient, *N* number

Research question 2.2 concentrated on the association between medication beliefs and the use of over-the-counter drugs (defined as drugs recommended by the pharmacists or bought in the supermarket without prescription). The Eta correlation coefficient showed no relevant correlation.

Likewise, no relevant correlation was detected between medication beliefs and the existence of a medication list (research question 2.3) nor the way the medication list was used by the participants (research question 2.4), i.e. whether it was updated regularly, shown when visiting a doctor or a pharmacy, taken along during emergencies or used as a reminder.

Research question 2.5 examined whether individual medication beliefs influenced the use of the tools provided by the project *MeinPlan*, that is, the website, the paper-based medication list template and the electronic medication list. No relevant correlations could be detected.

## Discussion

In this before-and-after study evaluating the project *MeinPlan* by means of representative surveys, no significant change in general medication beliefs among the target population could be shown. Equally, no statistical relationship between the population’s general medication beliefs and medication-related behaviour, i.e. neither the use of over-the-counter drugs/extensive polypharmacy, nor the active use of a medication list or the use of the tools offered by the project, could be determined.

These findings are partly in line with and partly contradictory to the results of other studies. BMQ values in a German primary care setting have shown similar values for general overuse (11.7 points) and general harm (8.7 points) [15]. Other studies have also found, that medication beliefs do not change over (short) time [16]. However, in a similar survey conducted in the Swedish general population, general medication beliefs were in contrast to our findings strongly associated with medication-use-patterns: Respondents using prescription and/or over-the-counter drugs reported stronger positive beliefs about the benefits of drugs in general compared to those who did not [17]. The authors concluded that addressing general beliefs about medication in patient counselling may be important – this is in line with our original assumption.

In another study, beliefs about the specific medication taken by the patient (but not beliefs about medication in general) were identified as a predictor for the use of over-the-counter analgesics [18]. Not receiving a medication list at discharge from hospital was associated with higher concerns towards the medication in patients with ischaemic heart disease [19]. In a German study among elderly patients with multiple morbidities and polypharmacy, regular receipt of an updated medication list was associated with a higher perceived necessity to take one’s specific medication. In addition, patients who found their medication list comprehensive had less concerns about their individual medication [20].

The results of this study have implications for the design of strategies intending to improve correct drug administration and the use of medication lists in the population. Our hypothesis was that addressing citizens’ general medication beliefs is a crucial element in reaching this goal. Most of our strategies intended to raise awareness of the risks of pharmacotherapy and the fundamental necessity of meticulous documentation and correct and skilful drug application. Additionally, we provided tools to enable patients to create a comprehensive medication list on their own. This approach is in accordance with the Health Belief Model (Fig. 1) in which perceived threats have a major influence on the likelihood of engaging in a health-promoting behaviour [12]. However, the results of this study indicate that general medication beliefs are not a decisive factor in that matter. The scores of the *BMQ General* did not differ between respondents who reported using a high number of (over-the-counter) drugs or a medication list and those who did not.

We decided to measure general beliefs because our campaign focussed on the general risks of polypharmacy. Within the scope of a large, population-based campaign it would have been difficult and ethically questionable to provide information about specific drugs or individual therapeutic regimes. Yet it is possible that our findings would have been different if we had used the BMQ Specific Scale which measures patients’ beliefs about the medications they actually take.

It is also possible that other aspects of the Health Belief Model, e.g. perceived benefits and barriers for the use of medication lists which we examined in another project [21] or socio-psychological factors are more relevant. Within the scope of large, population-based implementation programs such as *MeinPlan*, they could be addressed by designing a more benefit-oriented campaign instead of highlighting the risks of pharmacotherapy. It is possible that further strategies targeting individual barriers and involving social exchange, e.g. educational sessions in small groups or medication counselling in general practices and pharmacies, are necessary to actually change one’s individual behaviour.

The results of this study also indicate that the *cues to action* provided by the project *MeinPlan* were not successful in modifying medication beliefs which might be due to the fact that only a small section of the population could be reached by the programme: only 6% of the respondents stated to have heard about the project before participating in the survey. Consequently, if population-based campaigns are planned, sufficient resources to guarantee a successful and vast outreach of the measures have to be budgeted, e.g. involving radio, television, and social networks [22].

Our findings are especially relevant in Germany, where recently, after the start of our project, a federal standard medication list was introduced by law. Since October 2016 all patients taking at least 3 long-term drugs have the right to receive a medication list and physicians are obliged to issue the federal medication list in their practices [11]. Yet 1 year after the law came into force, the dissemination of the federal standard medication list was still poor: a survey among 324 health insured individuals showed that only 37% of those prescribed more than 3 drugs had received the federal standard medication list. Only half of them had been asked whether they were taking additional over-the-counter drugs, 43% had not received instructions on how to use the medication list and 21% had not been informed about the purpose and the administration of their medication [23]. Consequently, effective strategies to increase the active use of the federal standard medication list by health care professionals and patients need to be developed and applied.

### Strengths and limitations

The study was designed as an uncontrolled before-and-after study. Due to the absence of a control group, it remains unclear to which extent other factors not related to the intervention contributed to the fact that no effect of the campaign on medication beliefs could be shown.

The findings are based on a survey among a large, random sample which can be considered representative for the elderly German population at least in the Rhine-Neckar region. Even though the study used boosting methods to maximise response rates and prompted approximately half of the addressed audience to respond, there is still some inherent risk of selection bias [24].

The fact that international studies have shown a connection between medication beliefs and medication use pattern suggests that our findings are not necessarily transferable to other populations and countries.

While medication beliefs were measured by a validated instrument, all other items of the survey were self-developed. So, the reliability and validity of the phenomena measured by these items remain unclear.

## Conclusions

Our findings suggest that citizens’ general medication beliefs are not crucial for their decision to use a medication list or over-the-counter drugs. Future projects intending to increase correct drug administration and dissemination of medication lists should not concentrate on this aspect, especially not exclusively on risks and threats. Other factors, such as perceived benefits and barriers as well as socio-psychological factors might be more relevant and should be explored in future research.

## Data Availability

The datasets used and analysed during the current study are available from the corresponding author on reasonable request.

## Abbreviations

BMQ General: German version of the general part of the Beliefs About Medicines Questionnaire
CC: Correlation coefficient
SD: Standard deviation
N: Number
